# Enhancing Biocrust Development and Plant Growth through Inoculation of Desiccation-Tolerant Cyanobacteria in Different Textured Soils

**DOI:** 10.3390/microorganisms11102507

**Published:** 2023-10-07

**Authors:** Priya Yadav, Rahul Prasad Singh, Abeer Hashem, Elsayed Fathi Abd_Allah, Gustavo Santoyo, Ajay Kumar, Rajan Kumar Gupta

**Affiliations:** 1 Laboratory of Algal Research, Centre of Advanced Study in Botany, Institute of Science, Banaras Hindu University, Varanasi 221005, India; priya02061995@gmail.com (P.Y.); rah.singhbhu@gmail.com (R.P.S.); 2 Botany and Microbiology Department, College of Science, King Saud University, P.O. Box 2460, Riyadh 11451, Saudi Arabia; habeer@ksu.edu.sa (A.H.); 3 Plant Production Department, College of Food and Agricultural Sciences, King Saud University, P.O. Box 2460, Riyadh 11451, Saudi Arabia; eabdallah@ksu.edu.sa (E.F.A.); 4 Instituto de Investigaciones Químico Biológicas, Universidad Michoacana de San Nicolás de Hidalgo, Morelia 58030, Mexico; gustavo.santoyo@umich.mx (G.S.); 5 Amity Institute of Biotechnology, Amity University, Noida 201313, India

**Keywords:** *Nostoc calcicola* BOT1, *Scytonema* sp. BOT2, cyanobacteria, biocrusts, consortia, desiccation, revival potential

## Abstract

In recent years, there has been a burgeoning interest in the utilization of cyanobacteria for the purpose of land rehabilitation via enhancements in soil fertility, prevent erosion, and counter desertification. This study evaluated the ability of *Nostoc calcicola* BOT1, *Scytonema* sp. BOT2, and their consortia to form biocrusts on the substrate of coarse sand, fine sand, and loamy soil. A nutrient- and water-deficient substrate was inoculated with cyanobacteria to facilitate biocrust formation and evaluate their impact on agriculture. Cyanobacteria inoculation resulted in significant improvements in soil fertility, especially in coarse and fine sand, which initially had the lowest fertility. The findings of this investigation underscore that the consortium of cyanobacteria exhibited greater efficacy than individual strains in enhancing soil fertility and stimulating plant growth. The loamy soil treated with the consortium had the highest plant growth across all soil types, in contrast to the individual strains. The consortium of cyanobacteria showed promising results in promoting biocrust formation and fostering rice seedling growth in fine sand. This study provides empirical evidence supporting the potential utility of cyanobacterial consortia as a valuable tool for the rehabilitation of degraded land. Furthermore, the results indicate that cyanobacterial species can persist in soil environments even following prolonged periods of desiccation.

## 1. Introduction

Due to climate change, rising atmospheric carbon dioxide (CO_2_) concentrations are driving global changes in meteorological patterns and precipitation, which adversely affect plant growth patterns and also lead to soil degradation and loss of ecosystem services [1,2]. Cyanobacteria are often considered the initial colonizers of impoverished and degraded soils due to their remarkable ability to survive and thrive under challenging conditions, including high temperatures, prolonged exposure to UV radiation, and desiccation [3,4].

In recent decades, cyanobacterial inoculation has been considered a promising biotechnological approach to improve soil quality and crop yields. In general, this practice has been referred to as “cyanobacterization”, and plays a significant role in improving the soil quality by improving nitrogen (N_2_) content, and organic matter in the soil [5,6]. Furthermore, cyanobacterization has been successfully applied to control and reverse desertification [7]. However, restoring degraded arid soils under the current scenario of prolonged periods of drought and unpredictable rainfall patterns emerges as a big hurdle [8]. 

However, cyanobacterial inoculation has shown some remarkable positive results in maintaining the nutrient and moisture levels, soil stability, erosion resistance, and biodiversity of the soil [9]. This practice has also been used to promote the formation of biocrusts, which are important for soil stabilization and improved ecosystem functioning [10]. Biological soil crusts (BSCs) are intricate communities of microorganisms, including cyanobacteria, microalgae, mosses, and lichen [11], and are considered a significant component of arid and semiarid ecosystems, covering approximately 12% of the global land surface [12]. BSCs, which are formed by the binding of soil particles to an extracellular polysaccharide (EPS) secreted by cyanobacteria, play a crucial role in stabilizing sandy deserts and exert a significant influence on their biotic composition, covering about 70% of these regions [13,14]. 

The artificial establishment of BSCs through cyanobacteria inoculation is a promising biotechnological strategy for mitigating abiotic stress and maintaining soil quality and fertility [15,16]. This practice is grounded in the fundamental ecological functions of cyanobacteria in dryland ecosystems, where they are widespread and form complex communities [17]. Within biocrust communities, cyanobacteria play a vital role in shaping soil properties and functions through the methodology of soil aggregation, through binding soil particles together [18]. Cyanobacteria also have the ability to fix CO_2_, N_2_, and solubilize phosphate, leading to an increase in organic matter and nutrient content [19]. The utilization of cyanobacteria as bio-inoculants to ecologically restore degraded lands has been gaining attraction in recent years. Numerous investigations have demonstrated the positive effects of soil inoculation with cyanobacteria in various environments, showing promising results in enhancing soil conditions and promoting ecosystem recovery [6,20,21]. 

Cyanobacterial EPS plays an important role in the stabilization of dryland soils by forming stable organo-mineral layers. This is facilitated by the combined action of the cyanobacterial trichomes and EPS, which bind soil particles together and create a protective layer against desiccation and erosion [22]. Despite the promising results of cyanobacterial inoculation for desert restoration, there are still gaps in the knowledge and methodology that need to be addressed. For example, it is not yet fully understood how cyanobacteria respond to different environmental conditions, such as water availability. This knowledge is essential for developing effective restoration strategies that can ensure long-term success [23]. Cyanobacteria are remarkably adept at surviving desiccation and rapidly resuming photosynthetic activity upon rehydration. This ability is essential to their survival in the desert crusts, where they experience frequent periods of drought. Following rehydration, cyanobacteria are able to rapidly restore their metabolic activities and repair cellular components [24]. The selection of suitable inoculants for the restoration of desert areas is a critical process that requires careful evaluation of the cyanobacteria’s capacity to endure significant abiotic stresses. The availability of water is a key factor that influences the growth of cyanobacteria in semi-arid and arid regions, where they often encounter periods of drought [25]. Drought stress disrupts the structural organization of cyanobacterial cells, leading to impaired photosynthesis. This is because drought stress can damage the cell membranes, disrupt the thylakoid organization, denature proteins, and damage the deoxyribonucleic acid (DNA). As a result, the cell is unable to properly assimilate CO_2_ and produce energy [26,27]. Despite the challenges of desiccation stress, many cyanobacteria have evolved adaptive strategies to survive and even thrive in arid environments. When cyanobacteria desiccate, their metabolic systems shut down and they become inactive [28,29]. However, they can quickly resume their activity when rehydrated by water from dew, rain, or tidal flow [30].

The selection of suitable cyanobacterial species for biocrust restoration is a critical process and is based on a number of parameters, such as desiccation tolerance, ease of culture growth, EPS production, and resistance to challenging environments [6]. However, the ability to facilitate biocrust formation and enhance soil characteristics is considered an important characteristic. The selection of cyanobacterial species for biocrust restoration should also be influenced by the different types of soil. In addition, soil attributes such as mineralogy, texture, organic matter content, and nutrient levels also affect EPS production and growth. 

Although most studies on cyanobacteria inoculation have focused on sandy soils, many desert regions are characterized by fine-textured soils [31,32,33]. These soils are often prone to physical crusting, which can be exacerbated by the development of biocrusts, which positively affect the soil’s structure and hydraulic properties [34,35]. In previous studies, various authors have reported the inoculation efficacy of cyanobacterial species in improving the nitrogen and carbon content in the sandy soil. Additionally, previous studies have investigated the rehydration response of terrestrial desert crusts and sea-ice algae, but not desiccated agricultural field cyanobacterial crusts [10,36,37].

This laboratory-based study investigated the ability of N_2_-fixing cyanobacteria *N. calcicola* BOT1, *Scytonema* sp. BOT2, and their consortium to promote biocrust development, revival efficiency, and plant growth in three different soils. 

This is an important gap in the knowledge, as these crusts could potentially be used to improve soil quality and productivity. We hypothesized that these crusts would respond similarly to desert crusts, with the rapid reactivation of photosynthesis and migration of cyanobacteria to the surface of the mat [38,39]. The primary aim of this study was to access the ability of the selected cyanobacterial strain and its consortium to induce biocrust formation in three different types of soils (coarse sand, fine sand, and loamy soil) under nutrient-limited and water-stressed conditions. Further, we aimed to investigate the impact of cyanobacterial inoculation on biocrust growth, and assess the potential for revival after desiccation. In addition, we also scrutinized the effects of rehydrated cyanobacteria on the agronomic outcomes for rice seedlings.

## 2. Materials and Methods

### 2.1. Isolation of Cyanobacterial Species

Cyanobacterial strains were collected from different locations of agricultural land belonging to Banaras Hindu University, Varanasi, India. The collected cyanobacterial strains were then cultured on basal growth nitrate-free (BG-11 N^−^) agar plates to isolate heterocystous cyanobacteria [40]. The petri plates were incubated in a culture room at a controlled temperature of 28 ± 2 °C, with a light–dark cycle of 14 + 10 h and an artificial light intensity of 55 μ mol m^−2^/s. After colonization, the isolates were streaked onto the new agar plates and the process was repeated to isolate the pure strain.

### 2.2. Screening of Desiccation Tolerant Cyanobacterial Strain

#### 2.2.1. Revival Potential

The selection of desiccation-tolerant strain was made using the agar plates. All the isolated cyanobacterial strains were grown on the BG-11N^−^ agar plates for 60 days and then the plates were further dried under a laminar flow hood to ensure complete dryness. Dryness was assessed through a combination of weight measurements and visual inspection. After complete dryness was obtained, the desiccated cyanobacterial plates were sealed with parafilm and stored for 6 months at room temperature. 

Then, the desiccated cyanobacterial plates were rehydrated with 20 mL BG11 N^−^ medium for experimental purposes. The revival potential was analyzed by measuring the maximum photosynthetic quantum yield of photosystem II (Fv/Fm) using a pulse-amplitude modulation (PAM) (PAM-2500, Heinz Walz GmbH, Effeltrich, Germany). Fv/Fm is a widely used parameter to assess the efficiency of the photosynthetic apparatus in plants and algae. It provides insights into the health and vitality of photosynthetic organisms. Microscopy was performed after 96 h of rehydration to determine morphological changes that occurred during rehydration. 

#### 2.2.2. EPS Production

To elucidate the presence of EPS secreted by cyanobacteria, alcian blue dye was used to stain the algal strains, following the method outlined by Tamaru et al. [41] with slight modifications. In brief, 1 g of alcian blue was dissolved in 100 mL of deionized distilled water (DDW) containing a 3% acetic acid solution while maintaining a pH of 2.5. A 7 µL cyanobacterial sample was placed in the center of a glass slide, and 3 µL of alcian blue dye was directly applied to the sample. The sample was then incubated for from 5 to 7 min at room temperature. After incubation, the sample with the dye was covered with a glass coverslip, and any excess dye was removed by blotting with blotting paper. Subsequently, the slide was immediately observed under a light microscope.

### 2.3. Mass Cultivation and Experimental Design

The inoculum was prepared by culturing cyanobacteria in BG-11N^−^ medium in 2000 mL conical flasks. The cultures were harvested in the exponential growth phase through centrifugation and the pelleted biomass was washed twice with DDW to remove any remaining culture medium and EPS. The soils were oven-dried and autoclaved to suppress microbial activity. Each microcosm was created by filling 90 mm Petri plates with 50 g of autoclaved coarse sand, fine sand, or loamy soil (with particle sizes of 0.5–1 mm, 0.10–0.25 mm, and 0.002–0.05 mm, respectively). The pHs of coarse sand, fine sand, and loamy soil was 6.98, 7.44, and 7.68, respectively. The individual suspension of each inoculation treatment, which consisted of *N. calcicola* BOT1, *Scytonema* sp. BOT2, and a consortium of both strains in equal proportions, was poured on top of the substrate in the center of each microcosm. A total of 40 mL of DDW was poured into each microcosm, along with 10 mL of inoculum (chlorophyll-*a* concentration of 1.5 µg/mL), while for the consortium, 5 mL of each cyanobacterial strain was employed. The inoculated microcosms were incubated in a plexiglass growth chamber at the temperature of 28 ± 2 °C and the light-having condition of 55 μmol photons m^−2^/s was used for up to 40 days’ duration. No additional water was added to the microcosms during the experiment. Control microcosms were also incubated in the same conditions, but instead of cyanobacterial inoculation, DDW was used. After a span of 40 days, the crust was gathered from each microcosm using a spatula, and its thickness was subsequently assessed using a caliper. A total of 10 g of microcosm samples were collected from each petri plate and placed on pre-weighed aluminum foil paper to determine the water-holding capacity of the cyanobacteria. Subsequently, the petri plates were dried under a laminar flow hood. The dried plates were then stored in dry conditions for one year. After a year, the plates were rehydrated with 50 mL of DDW, and the revival potential of the cyanobacteria was measured after 12 h of rehydration. The plates were then inoculated with 50 rice seeds to evaluate the agronomic effects of desiccated cyanobacteria.

### 2.4. Estimation of Chlorophyll-a and EPS in the Biocrusts 

Chlorophyll-*a* (chl-*a*) and EPS were used as proxies for assessing biocrust development [42]. The extraction and quantification of chl-*a* followed the method of Castle et al. [43] with slight modification. In brief, 100 mg of biocrust samples were rehydrated with 5 mL of DDW for 12 h, then homogenized with 5 mL of ethanol at 80 °C for 2 h and incubated overnight at 4 °C. Further samples were centrifuged at 7000 rpm for 10 min and the absorbance of the supernatant was observed by UV-visible spectrophotometer at 665 nm and 750 nm. To eliminate any interference from residual scattering caused by the ethanol solution, the absorbance at 665 nm was corrected by subtracting the absorbance at 750 nm and the amended absorbance was used to evaluate the concentration of chl-*a* via the following equation:

Chl-*a* (µg/g) = [(11.9035 × A(665nm) × V)]/(mg crust × L)

The volume of the extract is represented by V, and L denotes the optical path length. 

The obtained results were reported in micrograms (mg) of chlorophyll per unit of soil dry weight. 

The quantification of EPS content was conducted using the anthrone method [44] with a slide modification, following the extraction procedure described by Rossi et al. [45]. First, the biocrust samples were homogenized in DDW with a 0.5 mM concentration of EDTA and incubated at 60 °C for 4 h. The samples were then centrifuged for 20 min at 5000 rpm, and the supernatants containing EPS were collected separately. To determine the total amount of EPS, 1 mL of each supernatant from the centrifuged crude homogenate was used. The anthrone reagent was prepared in 200 mL of chilled 95% H_2_SO_4_ by dissolving 400 mg of anthrone. The reaction mixture contained 1 mL supernatant and 4 mL of the freshly prepared anthron reagent. The mixture was then kept at room temperature for 15 min, followed by incubation in a preheated water bath for an additional 15 min. The reaction was halted by rapidly cooling the mixture with ice for 5 min. Further, the EPS was calculated by measuring the absorbance 625 nm using a UV-visible spectrophotometer. 

### 2.5. Rehydration Assay and Chlorophyll Fluorescence Measurements of Biocrust

Fv/Fm and ETR max were measured to determine the revival potential of the biocrust after 12 h of rehydration using PAM [46]. In brief, biocrusts of *N. calcicola* BOT1, *Scytonema* sp. BOT2, and a consortium with dry weights of approximately 100 mg were used for the experiment. They were then rehydrated in separate petri plates by adding 5 mL of DDW and, throughout the rehydration process, the biocrusts were kept under laboratory conditions. Further, before taking chlorophyll fluorescence measurements, the biocrusts were allowed to adapt to the darkness for at least 20 min.

### 2.6. Agronomic Effect of Revived Cyanobacterial Biocrust on Rice Seedlings

#### 2.6.1. Seed Germination, Root and Shoot Length 

The impact of rehydrated cyanobacteria on the seed germination, and other morphological yields was evaluated on the rice seeds (*Oryza sativa* var. Malviya 2.1), which were obtained from the Rice Breeding section, A.R.C. BHU, India. The seeds were surface-sterilized with ethanol and sodium hypochlorite, followed by a thorough rinsing with DDW. Further, 50 seeds were then placed in each of the petri dishes and the percentage of seed germination was measured after 48 h of incubation. The plant growth promotion was assessed through measuring the root and shoot length of rice seedlings after 15 days of growth. 

#### 2.6.2. Seedlings’ Dry Weight

Rice seedlings were subjected to an oven at a temperature of 60 °C, and their weight was measured until it reached a constant value. 

#### 2.6.3. Photosynthetic Pigments of Rice Seedlings

The estimation of photosynthetic pigments in rice seedlings was performed following the standard methods of Duxbury and Yentsch [47] and Maclachlan and Zalik [48], with slight modifications. Leaf pigments were extracted using acetone, and their absorbance at specific wavelengths (663, 645, 510, and 480 nm) was measured to determine chl-*a*, chlorophyll-*b* (chl-*b*), and carotenoid (carotene and xanthophyll) content, respectively. The calculations used were as follows:

Chl-*a* (mg/g) = [12.3A663 − 0.86A645] × V/[1000 × W × d]

Chl-*b* (mg/g) = [19.6A645 − 3.6A663] × V/[1000 × W × d]

Carotenoids (mg/g) = [7.6A480 − 1.49A510] × V/[1000 × W × d]

Total chlorophyll in leaf (mg/g) = Chl-*a* + Chl-*b*

Here, “W” represents the fresh weight of the sample in grams, “V” is the volume of the extract in milliliters, and d is the path length of light in centimeters.

#### 2.6.4. Flavonoids and Phenol Content of Rice Seedlings

To extract flavonoids and total phenolic content, rice seedlings were immersed in the 90% acetone solution. The total flavonoid content (TFC) of the sample was evaluated using the aluminum chloride (AlCl_3_) assay method following the standard protocol of Ordonez et al. [49]. In brief, 1 mL of the sample extract was mixed with 1 mL of 2% AlCl_3_, which was later mixed through agitation and kept for 2 h at room temperature. Further, the absorbance of the sample was recorded at 420 nm using a UV-Visible spectrophotometer.

The total phenol content (TPC) of the samples was measured using Folin–Ciocalteu reagent (FCR), following the standard protocol of Singleton et al. [50]. In brief, 0.5 mL of the supernatant was mixed with 1 mL of 2% Na_2_CO_3_ and 0.5 mL of 1N FCR. Subsequently, the volume was maintained to 5 mL using DDW. The mixture was further heated until it turned a blue color. Then, just after cooling to the normal room temperature, the absorbance was measured at 760 nm. 

### 2.7. Statistical Analysis 

Statistical analyses were performed using ANOVA with SPSS version 16. Tukey’s HSD test was used to determine significant differences between means at a significance level of *p* < 0.05. Graphs were generated using GraphPad Prism version 8.0.2.

## 3. Results and Discussion

### 3.1. Screening of Desiccation Tolerant Cyanobacterium

**Figure 1:**
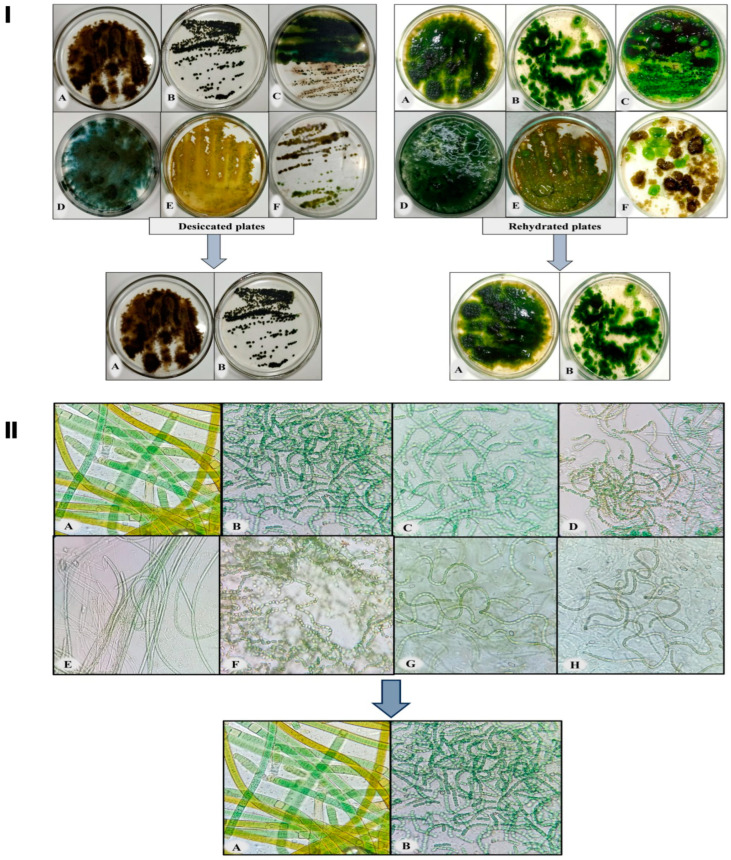
Screening of desiccation-tolerant cyanobacteria. **Panel I** represents the desiccated and rehydrated plates of the following cyanobacterial species: (**A**) *Scytonema* sp. BOT2, (**B**) *Nostoc calcicola* BOT1, Plate (**C**) contains two cyanobacteria: *Nostoc linckia* and *Nostoc* sp.1, (**D**) *Anabaena oryzae*, (**E**) *Nostoc* sp.2, and Plate (**F**) contains two cyanobacteria: *Nostoc spongiforme* (light green colony) and *Nostoc punctiforme* (brown colony). **Panel II** is the microscopic photographs of rehydrated cyanobacteria: (**A**) *Scytonema* sp. BOT2, (**B**) *Nostoc calcicola* BOT1, (**C**) *Nostoc linckia* (**D**) *Nostoc* sp.1, (**E**) *Anabaena oryzae*, (**F**) *Nostoc* sp.2, (**G**) *Nostoc spongiforme*, and (**H**) *Nostoc punctiforme*. Photography and microphotographs of these rehydrated plates were carried out 96 h subsequent to the commencement of the rehydration procedure.

**Figure 2:**
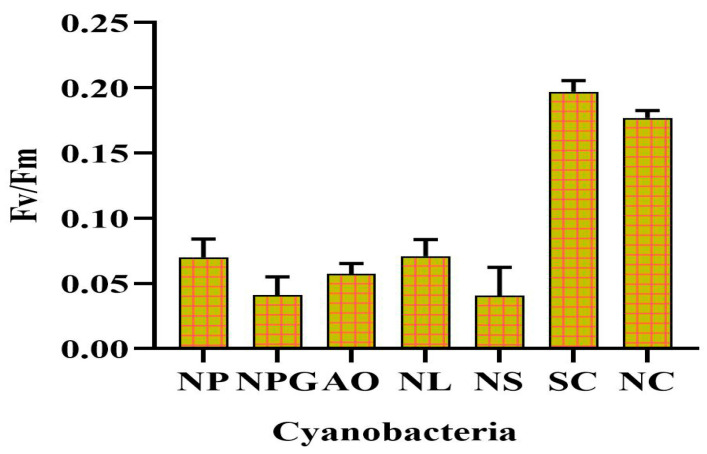
Revival potential of different cyanobacterial strains measured in terms of Fv/Fm after 12 h of rehydration. Fv/Fm is the maximum photosynthetic quantum yield of the PSII. In this context, **NP** (*Nostoc punctiforme*), **NPG** (*Nostoc spongiforme*), **AO** (*Anabaena oryzae*), **NL** (*Nostoc linckia*), **NS** (*Nostoc* sp.1), **NU** (*Nostoc* sp.2), **SC** (*Scytonema* sp. BOT2), and **NC** (*Nostoc calcicola* BOT1) represent the respective organisms.

Desiccation is a common stress that can negatively impact the growth and survival of cyanobacteria in semi-arid and arid regions. The selection of a desiccation-tolerant strain is a challenging task for researchers. Figure 1, panel I, illustrates the desiccated and revival state of various cyanobacterial species on agar plates, while in Figure 1, panel II, microscopic images of the screened cyanobacteria following 96 h of rehydration are depicted. *N. calcicola* BOT1 (Gene Bank Accession No. OP453348) and *Scytonema* sp. BOT2 (Gene Bank Accession No. MZ827168) were selected for further study based on their quick revival potential after 12 h of rehydration, as indicated by their high Fv/Fm values of 0.174 ± 0.01 and 0.196 ± 0.11, respectively, (Figure 2). The microscopic images of the two strains of cyanobacteria (Figure 1II) further support their revival potential. The images show that both strains were able to rapidly absorb water and rehydrate themselves by trapping the water in their sheaths. The sheaths of these two strains are highly effective at trapping water, which allows for them to revive quickly and efficiently. This is an important adaptation that helps these strains to survive in arid and semi-arid environments. These particular cyanobacterial species were selected for their widespread distribution in arid lands and various locations worldwide. This was based on previous studies that have shown these species to be tolerant of desiccation and other environmental stresses [51,52]. 

**Figure 3:**
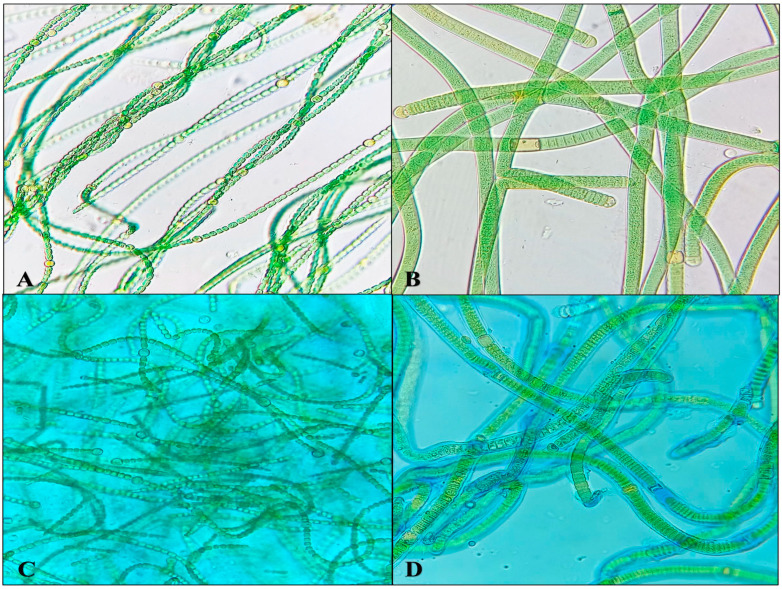
(**A**,**B**): Microphotographs of selected cyanobacteria *Nostoc calcicola* BOT1 and *Scytonema* sp. BOT2. (**C**,**D**): Microphotographs of *Nostoc calcicola* BOT1 and *Scytonema* sp. BOT2 with released EPS stained with alcian blue dye.

The production of EPS by cyanobacterial strains is regarded as a crucial characteristic. EPS serves to safeguard the photosynthetic apparatus from desiccation by retaining water molecules, thereby preserving its functionality. Two cyanobacterial strains, *N. calcicola* BOT1 and *Scytonema* sp. BOT2, were selected for the study as both strains secrete EPS. This was confirmed by staining the released EPS with alcian blue dye (Figure 3). 

### 3.2. Characteristics of the Biocrusts

**Figure 4:**
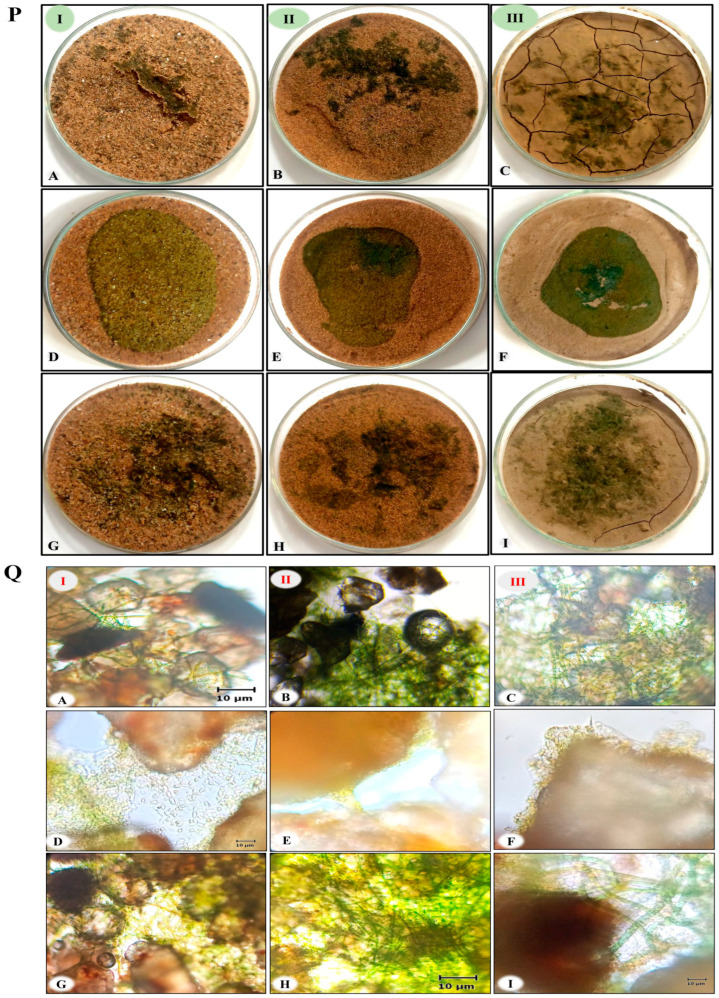
Panel **P** represents the biocrusts on different soil textures. **Panel Q** shows microscopic images of biocrust on different soil textures. (**I**–**III**) correspond to coarse sand, fine sand, and loamy soil, respectively. Images (**A**–**C**) depict the biocrust formed by *Scytonema* sp. BOT2. Images (**D**–**F**) show the biocrust formed by *Nostoc calcicola* BOT1. Images (**G**–**I**) display the biocrust formed by a consortium of *Nostoc calcicola* BOT1 and *Scytonema* sp. BOT2.

The inoculation of both cyanobacterial strains and their consortia resulted in the formation of biocrusts in all the tested soils. However, a very thin biocrust was formed on the surface of coarse sand, whereas a thicker crust emerged on loamy soils and fine sand. A thicker crust was formed on the loamy soil and fine sand due to the intermingling of cyanobacterial filaments with the compacted soil. The distribution of cyanobacterial cover on the soil surface varied between the two species. *N. calcicola* BOT1 displayed a more even dispersion, while *Scytonema* sp. BOT2 exhibited a patchy distribution and tended to create small biomass aggregates (Figure 4 panel P). The consortia, on the other hand, displayed maximum coverage over the soil surface and formed biocrusts on all soil types (Figure 4G–I in panel P). Under microscopic examination, distinct differences were evident between these two species (Figure 4A–I in panel Q). The consortia of cyanobacteria showed complete coverage with mats. The fine sand supported the consortium maximally, followed by loamy soil and coarse sand. Morphological observations of the top crust layer clearly revealed the presence of cyanobacterial filaments, which formed dense mesh-like structures (Figure 4G-I in pane Q). The filaments of *N. calcicola* BOT1 enveloped soil particles and created a complex interwoven network that resembled a blanket over the surface (Figure 4D–F in panel Q). In contrast, the thicker filaments of *Scytonema* sp. BOT2 clustered together in bunches amidst the soil particles, forming a structure reminiscent of a “coral reef” [53]. As expected, notable differences were also observed in the internal composition of the different soil types. The biocrust that developed on fine sand exhibited a more substantial structure. Specifically, *N. calcicola* BOT1 tended to encase soil particles within a dense layer of filaments, effectively binding the sand particles together (Figure 4D–F in panel Q). On the same substrate, *Scytonema* sp. BOT2 demonstrated a notably lower filament count than *N. calcicola* BOT1. The filaments of *Scytonema* sp. BOT2 ensnared and interconnected the sand grains, but they were not as densely packed as the filaments of *N. calcicola* BOT1 (Figure 4A–F in panel Q). The biocrust that formed on the coarse sand after the introduction of *N. calcicola* BOT1 was characterized by a uniform surface with a high density of filaments. In contrast, the biocrust that formed after the introduction of *Scytonema* sp. BOT2 was non-uniform and the filaments displayed lower density. The sheath materials of *N. calcicola* BOT1 served as cementing agents, holding the soil particles together. This resulted in the formation of a compact mat on the microcosm surface of all the different types of soil. In contrast, *Scytonema* sp. BOT2 did not form a compact mat on the microcosm surface of any soil type. A microscopic observation of the biocrust of *Scytonema* sp. BOT2 showed filaments that deeply penetrated the fine and coarse sand (Figure 4A–C). This may be a strategy allowing for *Scytonema* sp. BOT2 to tolerate more desiccation stress than *N. calcicola* BOT1. Notably, our results are significant because a biocrust formed without additional nutrients, soil-fixing agents, or superabsorbent chemicals. This is in contrast to previous studies that required the use of these materials [7]. However, in the recent past, various inoculation experiments were conducted without the addition of any nutrients and with a limited water supply [6,10]. These findings have implications for the economic and applied aspects of biocrust formation.

### 3.3. Estimation of Chlorophyll-a and EPS in the Biocrusts

**Figure 5:**
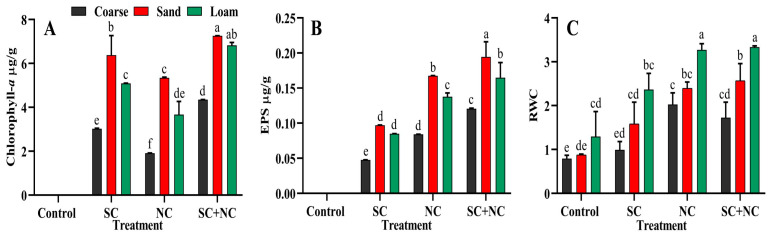
(**A**) Biocrust pigments, (**B**) EPS content and (**C**) relative water content (RWC) of different biocrusts. The data represent the means of three biological replicates, and the distinct letters on the bars indicate significance among the different cyanobacterial treatments. These data were considered significant at *p* < 0.05, as determined by Tukey’s post hoc test. The black, red, and green colors in the histogram indicate the soil types, while the letters on the *X*-axis represent the cyanobacterial treatments: **SC** for *Scytonema* sp. BOT2, **NC** for *Nostoc calcicola* BOT1, and **SC+NC** for the consortium of these two cyanobacteria.

The chl-*a* content of the biocrusts is presented in Figure 5A. The figure showed that fine sand supported maximum pigment levels, as compared to coarse sand and loamy soil, in all inoculation treatments. The consortia exhibited the highest chl-*a* content, followed by *Scytonema* sp. BOT2 and *N. calcicola* BOT1 inoculation. Fine sand with the consortium had the maximum chl-*a* content of 7.25 ± 0.01 µg/g, followed by *Scytonema* sp. BOT2 with 6.37 ± 0.89 µg/g and *N. calcicola* BOT1 with 5.34 ± 0.02 µg/g. The biocrust of *N. calcicola* BOT1 with coarse sand showed the minimum chl-*a* content of 1.91 ± 0.00 µg/g. Biocrusts formed on fine sand by *Scytonema* sp. BOT2 exhibited 1.19 times more chl-*a* content than biocrusts formed by *N. calcicola* BOT1. This is likely due to the presence of thick sheaths around the filaments of *Scytonema* sp. BOT2, which protect them from desiccation. In contrast, the chl-*a* content decreased during the incubation period, regardless of water availability. One possible explanation for this is that the extended period of desiccation followed by self-shading may contribute to the reduction in overall chl-*a* levels [33]. The inoculation of *Scytonema javanicum* and *Phormidium ambiguum* into burned sterilized soils induced the development of a cyanobacterial biocrust, which significantly improved the content of chlorophyll in comparison to the control soils [20].

Although the presence of water molecules in the biocrust significantly affects the amount of EPS (Figure 5B), the EPS content showed a slight accumulation in the core sand and the highest in fine sand. The overall accumulation of EPS in biocrusts suggests that cyanobacteria may allocate energy to EPS synthesis instead of growth. This is a potential strategy for enhancing resilience under adverse conditions, as EPS can help to protect cyanobacteria from desiccation and other environmental stresses [54]. The application of a consortia of cyanobacteria with fine sand showed promising results, enhancing EPS content (Figure 5B). The EPS content of the consortium was significantly higher than the EPS content of individual cyanobacterial strains, with a value of 0.194 ± 0.02 µg/g; this is 1.15 and 2.00 times greater than the EPS content of *N. calcicola* BOT1 and *Scytonema* sp. BOT2 in fine sand, respectively. In contrast, the EPS content of *Scytonema* sp. BOT2 was always minimum in coarse sand, with a value of 0.0476 ± 0.00 µg/g. These findings suggest that the consortia may be more effective than individual cyanobacterial strains at producing EPS under conditions of limited water availability. This is likely due to the synergistic interactions between the different cyanobacteria in the consortia. The consortia may also be more resilient to environmental stresses, as it has a wider range of adaptive strategies. The thickness of biocrusts tends to increase as they mature, which results in the enhanced physical stability of the soil. The formation of thick biocrusts in the consortia can be attributed to the unique ability of both cyanobacteria to move vertically in the sand (Figure 4G–I in panel Q). The strains *Scytonema* sp. BOT2 and *N. calcicola* BOT1 have different strategies for surviving desiccation. For example, *Scytonema* sp. BOT2 has a thick sheath that protects it from water loss, while *N. calcicola* BOT1 produces high amounts of EPS that bind the water molecules to the cells. The increased green color observed at the top crust when moisture availability is higher is indirect evidence of the gliding process, which is a way for cyanobacteria to obtain moisture [54]. Hormogones are small, motile filaments, generally shorter than the main filament, produced by some of the cyanobacterial strains to make them more mobile. The accumulation of filaments and cellular products in the deeper layers of the biocrusts may create a hydrophobic layer that repels water to protect the biocrust from erosion and to retain moisture. This water-repellency could be an evolutionary mechanism developed to adapt to dry environments, as it reduces water infiltration and slows down percolation through the soil, thereby increasing water availability in the top few millimeters. This may have been due to the production of EPS by the cyanobacteria, which helped to bind the sand particles together. It is also possible that some cells were sacrificed in order to maintain community viability and produce EPS.

During the measurement of water-holding capacity in terms of relative water content (RWC), which is directly related to particle size, loamy soil showed highest water-holding capacity. Loamy soil contains a mixture of sand, silt, and clay particles, which creates small pores that can hold water. In contrast, coarse sand has large pores that cannot hold as much water. The water-holding capacity of the consortium of cyanobacteria was significantly higher than the individual cyanobacterial strains and the control (Figure 5C). Consortium with loamy soil had 3.33 ± 0.02, followed by 3.21 ± 0.15, 2.36 ± 0.36, 2.15 ± 0.48 in *N. calcicola* BOT1, *Scytonema* sp. BOT2 and control, respectively. Fine sand had a moderate water-holding capacity of 2.57 ± 0.38, that is, 1.22, 1.42 and 1.62 times greater than *N. calcicola* BOT1, *Scytonema* sp. BOT2 and control.

EPS plays an important role in soil structure, functions and water-holding capacity [55]. This envelope serves to shield the cells from biological and physical stresses, ensuring their survival and well-being. It regulates the loss and uptake of water by the cells, protecting them from damage during the repeated cycles of drying and rehydration typical of arid environments [56,57]. EPS contributes to soil particle consolidation and overall soil stability and influences both microbial activity and the physical structure of the soil.

### 3.4. Revival Potential of Biocrust after Rehydration

**Figure 6:**
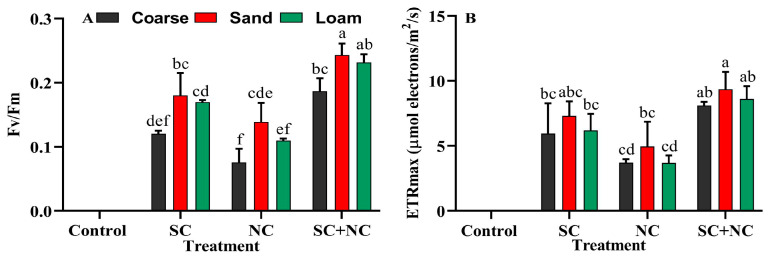
Recovery of biocrust after 12 h of rehydration. (**A**) Fv/Fm (maximum photosystem II quantum yield), and (**B**) ETRmax (maximum electron transfer rate). The data represent the means of three biological replicates, and the distinct letters on the bars indicate significance among the different cyanobacterial treatments. These data were considered significant at *p* < 0.05, as determined by Tukey’s post hoc test. The black, red, and green colors in the histogram indicate the soil types, while the letters on the X-axis represent the cyanobacterial treatments: **SC** for *Scytonema* sp. BOT2, **NC** for *Nostoc calcicola* BOT1, and **SC+NC** for the consortium of these two cyanobacteria.

Cyanobacteria are a prominent species within BSCs. Over their evolutionary trajectory, they have evolved specialized mechanisms to adapt to the ever-shifting environment, enabling it to endure frequent cycles of dehydration and rehydration. Remarkably, they can endure up to a 90% loss in cellular water and persist in a dormant state even under severe drought conditions. A brief episode of rainfall or even a light dew can swiftly revive them, causing them to regain their green coloration within minutes [58]. Due to their rapid recovery of photosynthesis upon rehydration, they serve as an excellent model for investigating the mechanisms behind the generation of photosynthetic machinery. Fv/Fm is a cumulative process that depends on the restoration of all parameters involved in electron transport in PSII. This suggests that the restoration of photochemical activity in PSII is a complex process that requires the coordinated function of multiple proteins and cofactors. The fluorescence signal contains valuable information, and parameters like the potential quantum yield of PS II. A notable augmentation in the Fv/Fm value was discerned within the consortium, in contrast with the individual cyanobacterial strains (Figure 6A). Remarkably, the Fv/Fm value demonstrated a consistent elevation in fine sand, followed by loamy soil, and exhibited the lowest levels in coarse sand. The maximum Fv/Fm was observed in consortium with fine sand: 0.24 ± 0.01, that is, 1.75 times greater than that of *N. calcicola* BOT1 and 1.34 times higher than that of *Scytonema* sp. BOT2. The minimum Fv/Fm was observed in coarse sand of *N. cacicola* BOT1, at 0.076 ± 0.02.

The maximum ETRmax was also found in the consortium with fine sand, at 9.35 ± 1.34 μmol electrons/m^2^/s (Figure 6B). The individual biocrusts of *N. calcicola* BOT1 and *Scytonema* sp. BOT2 with fine sand soil exhibited electron fluxes of only 4.95 ± 1.90 and 7.3 ± 1.13 μmol electrons/m^2^/s, respectively. In all the revival test, *N. calcicola* BOT1 showed the minimum Fv/Fm and ETRmax values in comparison to the consortium and *Scytonema* sp. BOT2. The chl-*a* content of biocrust is directly related to the Fv/Fm and ETRmax values after rehydration. The presence of high Fv/Fm and ETRmax values in *Scytonema* sp. BOT2 directly indicates that there are more viable filaments in the biocrust of all the different tested soils after desiccation. The differences observed between the cyanobacterial treatments can be attributed to the distinct morphological and adaptive characteristics of the different species. As an example, *Scytonema* sp. BOT2 is renowned for its distinctive sheath containing scytonemin, a photoprotective pigment that offers robust defense against desiccation and various environmental stressors. In contrast, *N. calcicola* BOT1 does not produce a sheath, making it more susceptible to desiccation and other environmental stresses [54]. Collectively, these findings indicate that the swift regeneration of photosynthetic pigments takes place upon rehydration in all the evaluated dried-out biocrusts.

### 3.5. Seed Germination, Seedlings Root–Shoot Length, and Dry Weight

**Figure 7:**
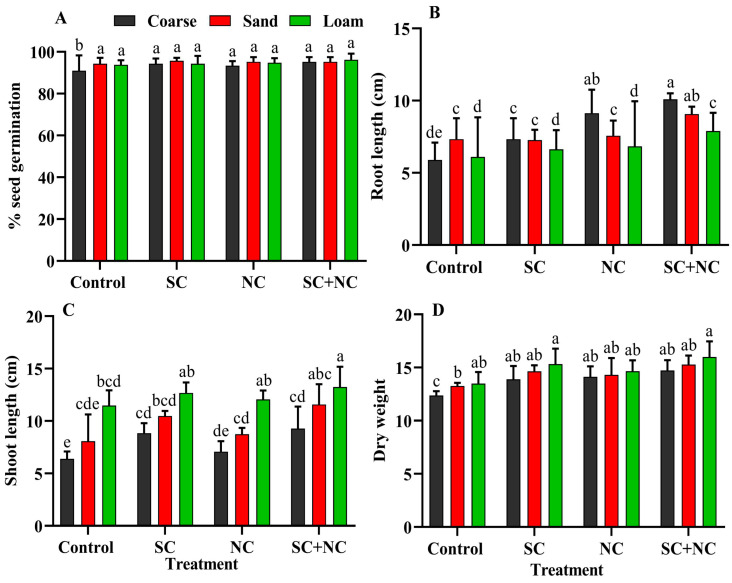
(**A**) Effect of different biocrusts on seed germination, (**B**) root length, (**C**) shoot length, and (**D**) dry weight of rice seedlings. The data represent the means of three biological replicates, and the distinct letters on the bars indicate significance among the different rice seedlings grown in soil treated with various cyanobacterial treatments. These data were considered significant at *p* < 0.05, as determined by Tukey’s post hoc test. The black, red, and green colors in the histogram indicate the soil types, while the letters on the X-axis represent the cyanobacterial treatments: **SC** for *Scytonema* sp. BOT2, **NC** for *Nostoc calcicola* BOT1, and **SC+NC** for the consortium of these two cyanobacteria.

This bioassay aimed to explore the role of the revived biocrust on the germination and growth of rice seedlings. The rice seeds were grown on a substrate consisting of coarse sand, fine sand, and loamy sand with a revived cyanobacterial biocrust such as *N. calcicola* BOT1, *Scytonema* sp. BOT2 and a consortium of these two cyanobacteria, without additional nutrients. A significant difference was found in all the soil types and between the various cyanobacterial treatments and the control. The consortium with loamy soil exhibited the highest seed germination percent of 96.19 ± 2.97%, followed by *Scytonema* sp. BOT2, 95.71 ± 3.77 and *N. calcicola* BOT1, 94.76 ± 2.18%. Figure 7A shows the significant difference between the control and treatment groups. Seed germination was minimal in control coarse sand, at only 90.95 ± 7.3%. In contrast, the germination rates were higher in the biocrust of loamy soil, the consortium (94.28 ± 1.42%), *N. calcicola* BOT1 (93.33 ± 2.47%), and *Scytonema* sp. BOT2 (94.22 ± 2.18%) biocrusts. Control soil without any cyanobacterial inoculation has minimum seed germination, and this may be due to the lack of nutrients. In a previous study, Yadav et al. [59] demonstrated that *N. calcicola* produces auxin in the medium but did not support seed germination. In recent studies, auxin showed positive effects on the seed dormancy, and was noted as a second phytohormone alongside ABA, which is responsible for inducing seed dormancy [60].

Regarding root length, the consortium and *N. calcicola* BOT1 biocrust with coarse soil showed the maximum root length, measuring 10.1 ± 0.4 and 9.13 ± 1.62 cm, respectively (Figure 7B). *Scytonema* sp. BOT2 biocrust with loamy soil achieved the minimum root length of 6.1 ± 2.74 cm. Uninoculated soils do not support increased root lengths, and the minimum length was observed in coarse sand, at 5.9 ± 1.2 cm. However the root length was found to be consistently higher in both fine and coarse sand compared to loamy soil, which may be attributed to the difference in porosity between these soil types (Figure 7B).

Shoot length was consistently highest in loamy soil, which was attributed to the presence of nutrients, while it was the lowest in coarse sand. Interestingly, the consortium plates exhibited the maximum shoot length in all soil types, with the highest being found in loamy soil. The maximum shoot length was recorded in consortium with loamy soil at 13.23 ± 1.94 cm, followed by *Scytonema* sp. BOT2, *N. calcicola* BOT1, and control, with 12.66 ± 1.00, 12.66 ± 0.84, 11.46 ± 1.45 cm, respectively (Figure 7C). The minimum shoot length was observed in coarse sand without inoculation, at 6.39 ± 0.70 cm. These findings demonstrate that the combination of fine sand and the consortium yields more favorable results compared with the use of individual cyanobacteria. Multiple studies have found that the phytohormones secreted by cyanobacteria play a significant role in increasing root length and dry weight, thereby improving nutrient uptake and overall plant growth [60,61]. The cell suspension of cyanobacteria on tobacco seed germination was found to not only enhancedgermination but also have a positive impact on callus differentiation [62].

The dry weight of rice seedlings was the highest in loamy soil across all treatments. Additionally, fine sand also supported seedling growth, with values of 15.23 ± 0.01 mg/plant in the consortium, 14.37 ± 0.01 mg/plant in *N. calcicola* BOT1, 14.60 ± 0.00 mg/plant in *Scytonema* sp. BOT2, and 13.02 ± 0.00 mg/plant in the control with fine sand (Figure 7D). The dry weight of rice seedlings in loamy soil with the consortium was 15.91 ± 0.01 mg/plant, which is 1.0, 1.04, and 1.17 times greater than that in *N. calcicola* BOT1, *Scytonema* sp. BOT2, and the control, respectively. The minimum growth was found in the control. In the control, N_2_-deficient plants typically exhibit growth inhibition, changes in pigment content, and a reduced dry weight. The findings suggest that the plants utilized nitrogen and carbon nutrients released by the N_2_-fixing cyanobacteria. In addition to supporting nutrient acquisition, cyanobacteria have been shown to positively influence germination percentage, growth, and, ultimately, yield in plants [63]. In the context of rice cultivation, its enhanced tolerance to salinity has been validated through the introduction of strains isolated from saline soils, including *Anabaena variabilis, N. calcicola*, and *N. linkia*. This approach led to an augmentation in root length, as observed by El Sheek et al. [64], subsequently promoting both seedling growth and yield, akin to the effects of *Nostoc commune* and *Nostoc carneum*, as demonstrated by Chittapun et al. [65]. The application of Gibberellin-like plant growth regulators isolated from the *Scytonema hofmanni* significantly enhanced the level of carotenoids and dry weight of shoots [66]. Furthermore, in the case of wheat and millet, the utilization of a consortium comprising *N. punctiforme* and *Nostoc ellipsosporum* demonstrated improvements in the microbial activity, nutrient status, and physical structure of salt-affected soils. This resulted in a substantial increase in growth and yield, as evidenced by the research of Nisha et al. [67].

### 3.6. Effect of Revived Cyanobacterial Biocrust on Growth of Rice Seedling

**Figure 8:**
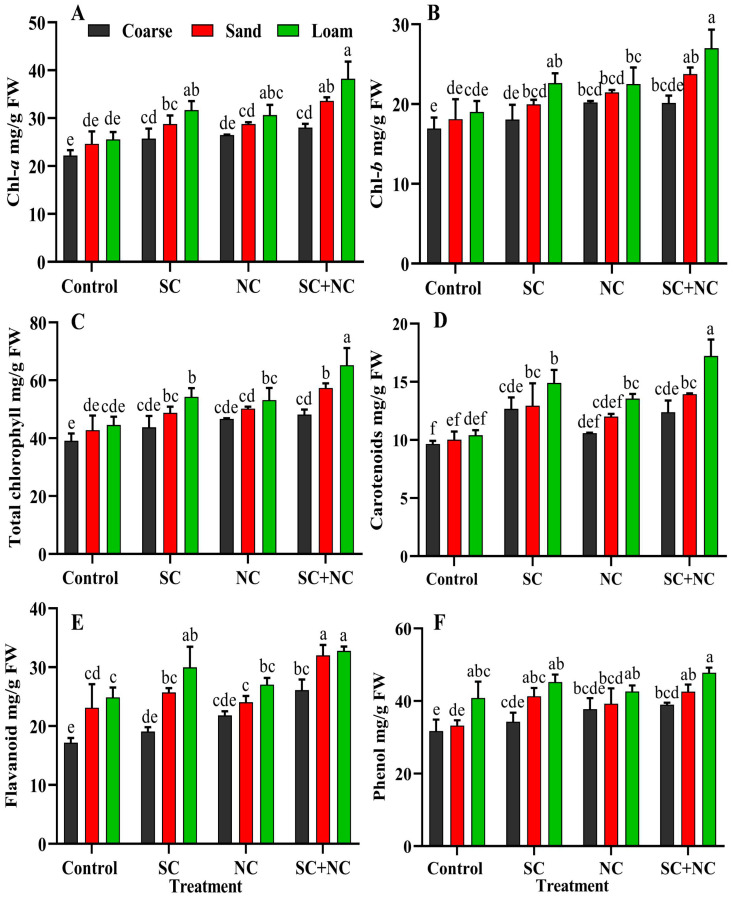
Illustrates the impact of various biocrusts on both pigments and non-enzymatic antioxidants in rice seedlings after 15 days of growth. (**A**) Chlorophyll-*a*, (**B**) chlorophyll-*b*, (**C**) total chlorophyll, (**D**) carotenoids, (**E**) total flavonoid content (TFC), and (**F**) total phenolic content (TPC). The data represent the means of three biological replicates, and the distinct letters on the bars indicate significance among the different rice seedlings grown in soil treated with various cyanobacterial treatments. These data were considered significant at *p* < 0.05, as determined by Tukey’s post hoc test. The black, red, and green colors in the histogram indicate the soil types, while the letters on the X-axis represent the cyanobacterial treatments: **SC** for *Scytonema* sp. BOT2, **NC** for *Nostoc calcicola* BOT1, and **SC+NC** for the consortium of these two cyanobacteria.

This experiment was conducted to investigate the impact of the nutrient and fertilizing potential of revitalized biocrusts. In semi-arid and arid regions, there is a demand for biological fertilizers, as large quantities of traditional fertilizers are required to support plant growth. To address this issue, four criteria are essential: the ability to thrive in limited water and low-nutrient conditions, the ability to fix essential nutrients like carbon and nitrogen through processes like photosynthesis and N_2_-fixation, and a quick revival ability [68]. *N. calcicola* BOT1 and *Scytonema* sp. BOT2 possess these qualities. To ensure accurate results and avoid the influence of other organisms, a bioassay test was conducted using a monoculture of *N. calcicola* BOT1, *Scytonema* sp. BOT2, and their consortium. This study represents the first instance of using N_2_-fixing *N. calcicola* BOT1, *Scytonema* sp. BOT2, and their consortium to investigate plant growth after revival. The results revealed that the presence of the cyanobacterial mat enhanced rice seedling growth in all soil types. Individual cyanobacterial strains also promote the growth of rice seedlings in all soil types, while consortia with loamy soil supported the maximum growth in terms of total pigment and dry weight. However, the maximum photosynthetic pigment in rice seedlings was found in consortium with loamy soil. In loamy soil, chl-*a* content was measured at 38.24 ± 1.23 mg/g FW in the consortium, 30.63 ± 0.123 mg/g FW in *N. calcicola* BOT1, 31.68 ± 0.35 mg/g FW in *Scytonema* sp. BOT2, and 25.54 ± 0.19 mg/g FW in the control (Figure 8A). The maximum growth of rice seedlings was observed in the biocrust as compared to control in all treatments. Loamy soil supports growth; this may be due to presence of nutrients. Although, during the study, coarse and fine sand also promoted seedling growth, this growth may be supported by the biocrust (Figure 7A–D). Chl-*b* exhibited a pattern similar to that of chl-*a*, with its highest concentration observed in the consortium grown in loamy soil (Figure 8B). Biocrust with loamy soil shows total chlorophyll of 65.26 ± 1.07, 53.13 ± 0.3, and 54.33 ± 0.17 mg/g FW in consortium, *N. calcicola* BOT1 and *Scytonema* sp. BOT2, respectively (Figure 8C). Carotenoids are accessory pigments that act as antenna pigments and also follow a similar trend to chlorophyll pigments; the maximum concentration was found in loamy soil after all the different treatments. In addition to consortia, the biocrust with *Scytonema* sp. BOT2 enhanced carotenoids in all soil types (Figure 8D). The results were 12.67 ± 1.09, 12.94 ± 0.19 and 14.91 ± 0.51 mg/g FW in coarse sand, fine sand and loamy soil. According to Katoh et al. [62], when *Nostoc* sp. and *Scytonema* sp. were applied, they led to an enhancement in the nitrogen and carbon content of the soil, which, in turn, promoted ion uptake and plant growth. The researchers believed that micro- and macronutrients play a major role in supporting plant growth, and cyanobacteria play a significant role in supplying these nutrients.

### 3.7. Phenol and Flavonoid Content of Rice Seedlings

The biocrusts of different cyanobacteria promote the TFC and TPC of rice seedlings and the maximum concentrations were always found in the biocrust formed by consortia (Figure 8E,F). TFC and TPC follow a similar pattern to pigment and dry weight. TFC was 32.77 ± 0.75, 27.03 ± 1.15, 29.99 ± 3.48, and 24.89 ± 1.66 mg/g FW in consortium, *N. calcicola* BOT1, *Scytonema* sp. BOT2 and control, respectively. Biocrust with fine sand have 32.02 ± 1.74 mg/g FW in the consortium, which is 1.33, 1.24 and 1.38 times greater than *N. calcicola* BOT1, *Scytonema* sp. BOT2 and control (Figure 8E). The TPC of rice seedlings reached the maximum in the consortium with loamy soil, at 47.77 ± 1.14 mg/g FW. The consortium with fine sand contained 42.56 ± 0.19 mg/g FW, which is 1.08, 1.03 and 1.28 times greater than *N. calcicola* BOT1, *Scytonema* sp. BOT2 and control (Figure 8F). Coarse sand contained the lowest TFC and TPC content of rice seedlings in all treatments. These findings highlight the beneficial effects of cyanobacteria in promoting plant growth and enhancing the soil environment for better plant development. Existing evidence supports the notion that when plant growth-promoting rhizobacteria (PGPRs) are introduced, they release a variety of secondary metabolites that act as growth-promoting agents [69], as well as signaling molecules in the rhizosphere that enhance plant growth [70]. Flavonoids play a significant role in plant–microbe interactions and contribute to the enhanced colonization of roots by microbes [71]. Additionally, they promote allelochemical effects on the population of other organisms [72] and serve as signal molecules [73]. A substantial increase in phenolic accumulation was observed in rice leaves compared to the control group after inoculation of cyanobacterial organisms [74,75].

## 4. Implications for Soil Restoration

EPS produced by cyanobacteria can bind together with sand grains, creating a cohesive and stable layer [33]. This layer helps to reduce soil erosion, which is a significant factor contributing to land degradation in drylands [76,77]. Studies in China have shown that the inoculation of sand dunes with the cyanobacteria *Microcoleus vaginatus* and *S. javanicum* can successfully stabilize the soil and promote the colonization of other biocrust organisms. The reason behind this is the EPS production of cyanobacteria, which binds the sand grains together and helps to retain moisture [7,9]. The EPS’ also provide a substrate for other organisms to colonize, which further enhances the stability and productivity of the biocrust [56,78]. This is likely due to the higher growth and release of EPS by the consortium compared to the individual cyanobacteria *N. calcocola* BOT1 and *Scytonema* sp. BOT2. In a previous study, sandy soil inoculated with the cyanobacteria *Phormedium ambiguum* exhibited increased hydrophobicity, possibly due to the reduction in EPS produced under more water-stressed conditions [57]. Furthermore, inoculation with consortium resulted in an increase in EPS in all three studied soils compared to other inoculation treatments. Our findings are consistent with previous studies that have shown that inoculation with cyanobacteria can lead to increased colonization rates and chl-*a* concentration in arid soils. For example, a study by Román et al. [79] found that soils inoculated with *Nostoc* and *Scytonema* strains from arid soils in Spain had higher colonization rates than uninoculated soils. Similarly, our study found that the consortium inoculation treatment resulted in a significantly higher chl-*a* and EPS content than the individual treatments for all soil types. In their studies, Wang et al. [10] combined species from the *Scytonema* and *Microcoleus* genera, while Muñoz-Rojas et al. [21] utilized a mixture of *Tolypothrix, Nostoc*, and *Scytonema*; the results of both studies indicated that these combinations were effective in forming biocrusts.

*Scytonema* sp. BOT2 has several morphological features that demonstrated effectivity in soil colonization. In addition to its scytonemin, which contains an exopolysaccharide sheath, it has large, disk-shaped cells that play a significant role in the formation of extensive mats of trichomes. These features allow for *Scytonema* sp. BOT2 to spread easily among soil particles and to withstand desiccation and other environmental stresses [53,80]. The cells of *Scytonema* sp. BOT2 are large and disk-shaped, which provide them with a higher surface-area-to-volume ratio. This allows the strain to absorb more water and nutrients from the soil. The combination of these morphological features makes *Scytonema* sp. BOT2 an effective biocrust-former and a valuable tool for soil restoration and rehabilitation. The combination of salinity tolerance and EPS production properties of *N. calcicola* make it a valuable tool for soil restoration in saline environments. Further, it showed an ability to colonize the soil efficiently and also helps to improve the soil quality by binding soil particles together, preventing erosion and providing a substrate to the other organisms that helps with colonization [81,82]. The morphological and physiological characteristics of both *Scytonema* sp. BOT2 and *N. calcicola* BOT1 may have contributed to their relatively high performance in terms of the colonization of soils. *Scytonema* sp. BOT2 has a heavy, pigmented sheath that provides protection against desiccation, and *N. calcicola* BOT1 produces large amounts of EPS, which help to bind soil particles together and improve soil texture. The combination of these characteristics made the consortium more resistant to desiccation and more effective at colonizing soils.

## 5. Conclusions

In summary, our findings demonstrated the successful screening of desiccation-tolerant cyanobacterial strains. The introduction of these strains to soil resulted in the formation of biocrusts across various soil types. The consortium of *N. calcicola* BOT1 and *Scytonema* sp. BOT2 exhibited a higher capacity for promoting biocrust formation and increasing EPS content in all soil types. The results exhibited significant disparities depending on the species and soil texture. Notably, the introduction of *N. calcicola* BOT1 had a more pronounced impact on the EPS content. Biocrusts were successfully revived after one year of desiccation. Among the different inoculation treatments, the consortium exhibited the most favorable outcomes and promoted the growth of rice seedlings in all soil types. Both the consortium and *Scytonema* sp. BOT2 biocrusts exhibited a more pronounced effect on the germination and growth of rice seedlings. These findings highlight the importance of carefully selecting cyanobacterial species for soil inoculation based on their primary functional roles and compatibility with specific soil conditions. The consideration of functional roles of cyanobacterial species and their compatibility with specific soil conditions is crucial to maximize the positive impacts on soil quality. Further research is needed to explore the feasibility of cyanobacterial inoculation in various ecosystems with distinct soil types and climate conditions. This research is essential for developing a comprehensive, adaptable, and effective decision-making framework for restoring arid lands by promoting biocrust formation. The collective results of the study suggest that a cyanobacterial consortium could serve as a valuable alternative to enhance soil fertility, offering a sustainable and efficient solution for promoting plant growth in challenging environments.

## Data Availability

The datasets used and analyzed during this study are available from the corresponding authors on reasonable request.

## Abbreviations

BSCs: Biological soil crusts; EPS: extracellular polysaccharide; BG: basal growth; DDW: deionized distilled water; PAM: pulse-amplitude modulation; PSII: photosystem II; Fv/Fm: maximum photosynthetic quantum yield of the PSII; TFC: total flavonoid content; TPC: total phenol content; FCR: folin-ciocalteu reagent; FW: fresh weight; PGPRs: plant growth-promoting rhizobacteria.
